# Real-time visual interactions across the boundary of awareness

**DOI:** 10.1038/s41598-018-24554-1

**Published:** 2018-04-24

**Authors:** Noya Meital-Kfir, Dov Sagi

**Affiliations:** Department of Neurobiology, Weizmann Institute of Science, Rehovot, 76100 Israel

## Abstract

Recently, using Motion Induced-Blindness (MIB), we have shown that two visual stimuli, one consciously experienced and one not, interact as a function of feature and object similarity, pointing to preserved visual representations of objects, and their constitutive features, in the absence of perceptual awareness. Here we investigated whether these representations preserve the memory of the previously perceived stimulus by testing interactions with the unperceived stimulus modified while it is invisible. Observers performed the MIB task, wherein an object ‘Target’ (a plaid object) was morphed into one of its features (an oriented Gabor patch) once its disappearance was reported. Reappearances of the morphed target were induced by a visible ‘Cue’ (object or feature), with reappearance frequency used to quantify the interaction between the visible cue and the invisible target. Reappearance rates were highest when the morphed target and the cue shared the same orientations, with the plaid-cue showing reappearance rates equal to that of the orthogonal-cue. Our findings indicate that target-cue interactions do not depend on memory-stored representations, but rather, on the current state of the consciously unavailable target. We suggest that visual objects can be constructed and deconstructed in the absence of conscious perception, but only objects are consciously available.

## Introduction

Salient visual stimuli can disappear and reappear from conscious awareness when surrounded by a high-contrast moving background, a phenomenon known as Motion-Induced Blindness^1^ (MIB; www.scholarpedia.org/article/Motion_induced_blindness). MIB disappearances are an all-or-none phenomenon and can last up to several seconds, even with a high-contrast target located near fixation^2^. It has been proposed that known low-level processes such as contrast adaptation^3^, and filling-in^4^ cannot completely account for MIB, and that competition between the neural representations of the static target and the moving background needs to be considered in order to explain the range of effects found^1,2,5^.

Here we attempted to explore the nature of stimulus representations in our visual system while the corresponding stimulus is outside of awareness, that is, it is not available to subjective experience. Of particular interest are the defining features of these representations, potentially allowing us to draw conclusions regarding the distinctive features of subjective experience, and its function. The all-or-none nature of MIB disappearances serves as a tool to test visual processing at times of perceptual suppression. Indeed, some studies have shown that low-level adaptation^6,7^ and grouping by the Gestalt principles^1,8^ are maintained during MIB episodes. In a more recent study we attempted to specify the information available in perceptually invisible stimuli^9^. Specifically, we investigated whether invisible stimuli are represented as a collection of features or as integrated objects. Using a method introduced by Kawabe *et al*.,^10^ we measured the reappearances of a perceptually invisible ‘Target’ in the presence of a visible ‘Cue’. Interestingly, our results indicated that the cue interacts with the suppressed target as a function of orientation similarity (~30° bandwidth) and distance (~1° range)^9^. We suggested that feature-specific interactions between the seen and the unseen imply that information related to the unseen target is available in the system, guiding interactions with the cue^9^. An object-based representation in the absence of conscious experience was tested by examining the interaction between a compound stimulus, composed of two orthogonal orientations, and its features. Importantly, we showed that asymmetric relations exist between aware and unaware object representations. A visible plaid cue did not effectively interact with a target defined by only one of its components; however, an invisible plaid target efficiently reappeared with component cues and a plaid target. It was suggested that perceptually visible as well as invisible objects are represented by combinations of features, possibly at the object level of processing. However, only the perceptually invisible ones are decomposed into their constitutive features^9^.

Our previous findings^9^ indicate similarity-based interactions between the perceived and the unperceived ones, pointing to object-based representation in the absence of awareness. However, it is not yet clear whether the observed target-cue interactions taking place during perceptual suppression indicate interactions across awareness states. It is possible that the interactions are affected by the available memory of the target, and thus preserve target-related representations available before suppression. To address this issue, we tested the interactions between a visible cue and an invisible target modified during its disappearance, and investigated whether the interactions depend on the actual representation of the invisible target during the time of suppression. A real-time interaction would suggest that interactions occur across the awareness boundary. Furthermore, it would imply that interactions are dynamic and sensitive to real-time changes in the visual representations, even in the absence of perceptual feedback.

## Methods

### Observers

The experiments included 10 untrained naïve observers with normal or corrected-to-normal vision. Observers were excluded from the experiment after the first experimental session (see below, Task & Procedure) if their overall reappearance rate was <20% or >99% (N = 3). Seven observers (5 females, aged 23–27) completed all the experimental sessions and were included in the analyses. This sample size was in accordance with our previous results^9^. Observers gave their written informed consent and were paid for participating. The procedures were in accordance with the Declaration of Helsinki and were approved by the Review Board of the Weizmann Institute.

### Apparatus

The experiments were carried out using the MATLAB Psychophysics toolbox (Psychtoolbox-3; www.psychtoolbox.org;)^11^. Stimuli were displayed on a gamma-corrected 23.6” VIEWPixx/3D monitor (1920 × 1080, 10 bit, 120 Hz) viewed at a distance of 100 cm in an otherwise dark room.

### Stimuli

The target was a plaid pattern composed of −45° and +45° Gabor patches (σ = 0.12°, ω = 5.67cpd). Each component had a contrast of 50% before its disappearance was reported. After its disappearance was reported, the target was morphed into one of its components (a Gabor patch, either +45° or −45°) by reducing the contrast of the other plaid components using a logistic decay of 150 ms (see next Tasks & Procedure). Cues (50% contrast) were either −45° or +45° Gabor patches, or a plaid pattern composed of the sum of the two orientations (the same as the target). The Target was presented in the upper left quadrant at an eccentricity of 1.5° to a central fixation point (0.31° diameter). The cue was eccentric to the target at a distance of 0.7° from the target’s center. Target and cue were placed within a mask composed of 10 × 10 black ‘+’ patterns (0.7° width, 1.4° spacing). The complete mask configuration was rotated clockwise, at 2.4 sec/cycle, around the central fixation point. Background-colored “protection zones” (0.88° and 0.62° diameter, respectively), around the target and the cue locations were used to avoid overlap between the stimuli and the mask. Stimuli were displayed over a gray background (mean luminance: 48 cd/m^2^).

### Tasks and procedures

**Figure 1:**
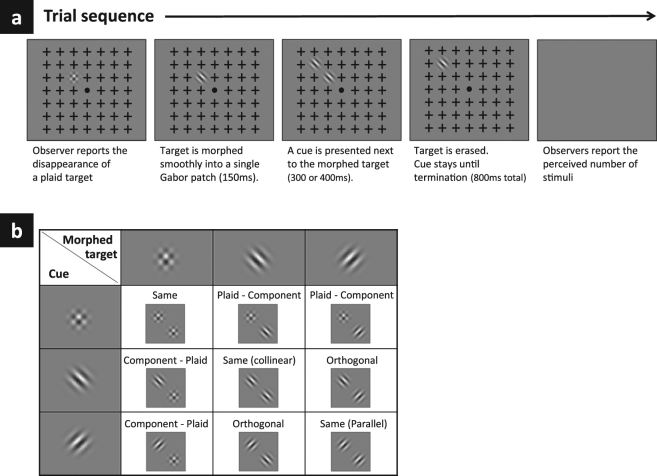
Experimental paradigm; a single trial (**a**). All possible combinations of a morphed target and cue (**b**).

There were four daily sessions: one familiarization session and three experimental sessions. The familiarization session was designed to allow for some experience with the MIB phenomenon^1^; observers performed a standard MIB paradigm with a single Gabor patch as the target, which was removed after its disappearance was reported with a variable delay time^9^ (200, 300, 400, or 600 ms). At the end of each trial, the observers reported the number of stimuli they had perceived from the time when the disappearance was reported (either 0 or 1 stimulus). The methods used in the experimental sessions were similar to those used in our previous study^9^ but with several important modifications. The trial began by presenting the MIB display (i.e., target and mask; Fig. 1a). The observers fixated on a dot at the display’s center and pressed a button to report the disappearance of a plaid target. After the disappearance was reported, the target was morphed into one of its components (see Stimuli, above). A ‘Cue’ was presented next to the morphed target (150 ms delay relative to disappearance), producing nine possible combinations of target and cue (Fig. 1b). The morphed target and the cue were presented concurrently for a limited interval of 300 or 400 ms before the morphed target was smoothly erased (a logistic decay of 100 ms). These two time windows were chosen based on prior experiments, which indicate an optimal time window for reappearances; the frequencies of reappearances became saturated above 500–600 ms and decreased below chance level at 200 ms^9^. The Cue was presented until the trial was terminated (800 ms total). Observers were instructed to report the number of stimuli they had perceived from the time of target disappearance (i.e., zero, one, or two patches). A report of two perceived stimuli, following suppression, indicated the induced reappearance of the target by the cue. The following trial was initiated by the observer pressing a key. The shape of the cue (+45°, −45°, plaid) was randomized within a session. Each session included cue-trials in which the target remained a plaid pattern (no morph condition) and control trials without a cue (spontaneous reappearances). Overall, each participant completed 30 trials with each morph type × cue × time-window combination during three experimental sessions (45 minutes each).

### Data analyses

The frequencies of target reappearances induced by the cue were calculated for each observer as the percentage of trials in which the observer reported two perceived stimuli, from the total number of trials. The SPSS 21 package was used for all statistical analyses (IBM Corp. Released 2012. IBM SPSS Statistics for Windows, Version 21.0. Armonk, NY: IBM Corp.). Repeated-measures ANOVAs were used to test the differences in the frequency of reappearances across conditions. Sphericity violations were corrected by the Greenhouse-Geisser method. Paired-samples t-tests were used for comparing the differences between two data points.

## Results

**Figure 2:**
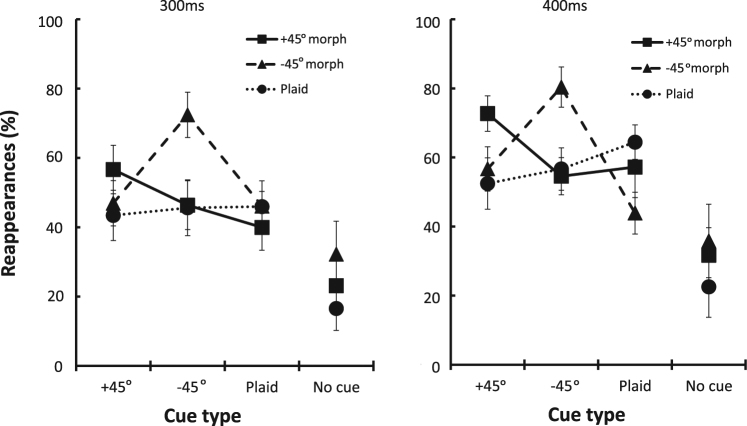
The frequencies of target reappearances as a function of morph and cue type at the 300 ms and 400 ms time windows. Datum points represent means across observers (N = 7), and error bars represent the standard error of the mean.

To determine whether the interactions depend on the actual stimulus presented during perceptual suppression, the target was modified right after its perceptual disappearance and a cue was presented next to it. Observers reported the number of perceived stimuli from the time of target disappearance (i.e., zero, one, or two stimuli). A report of two perceived stimuli, following suppression, indicated an induced reappearance of the target by the cue. Importantly, observers did not report ‘zero’ in ‘cue’ trials (thus, cues were always detected), and they did not report more than one stimulus in the ‘no cue’ trials (control trials). Figure 2 illustrates the frequencies of reappearances as a function of morph and cue type at the two time windows, showing the dependence of the cue effect on the morph condition. Of particular interest is the increased rate of reappearances when the cue and the morphed target were at the same orientation, that is, the −45° and +45° morph curves show the highest rate of reappearance at their corresponding cue orientation (Fig. 2).

Analysis of the time to disappearances (i.e., visibility duration) showed a distribution similar to that reported in previous MIB papers^2,5^ (Mean = 6.95 sec, SD = 3.96, Median = 5.12). Overall, reappearances induced by the Gabor cues were more frequent than were reappearances without a cue (i.e., spontaneous reappearances) (*t*(6) = 3.54, *p* = 0.025 and *t*(6) = 2.97, *p* = 0.012, for −45° and +45° cue, respectively). However, over all target types, there were no observed differences in the reappearance rates of no-cue trials and plaid-cue trials (*t*(6) = 2.19, *ns.)*. The effectiveness of the cue was found to depend on the specific target shown during the perceptual suppression. Rm-anova with two within-subject factors (3 × 3) displayed a significant morph × cue interaction (*F* (4, 24) = 14.58, *p* < 0.0001, η^2^_p_ = 0.7 and *F* (4, 24) = 15.02, *p* < 0.0001, η^2^_p_ = 0.71, for 300 and 400 ms, respectively). Complementary post-hoc analyses indicated that the frequencies of the reappearances of the morphed Gabor targets were highest in the presence of cues having the same orientation; a −45° morphed target reappeared more frequently in the presence of a same-orientation (collinear) cue (−45°) than in the presence of a plaid cue (300 ms: *t*(6) = 3.12, *p* = 0.02; 400 ms: *t*(6) = 7.68, *p* < 0.001), whereas for the +45° morphed target, the same-orientation (parallel) cue (+45°) induced higher frequencies of reappearances relative to a plaid cue (300 ms: *t*(6) = 3.68, *p* = 0.01; 400 ms: *t*(6) = 2.43, *p* = 0.05). Furthermore, no significant differences existed between orthogonal cues and the plaid cue (300 ms: *t*(6) = −0.17, *ns. and t*(6) = 1.25, *ns; 400 ms: t*(6) = −1.5, *ns. and t*(6) = −0.42, *ns*., for −45° and +45° morphed targets, respectively).

In line with our previous findings, indicating that unperceived objects are represented at multiple levels of abstraction, the reappearances of a morphed plaid target were not significantly affected by cue type (*F* (2, 12) = 0.89, *ns*. and *F* (2,12) = 3.25, *ns*., for 300 and 400 ms, respectively).

Last, analysis of the time window indicated increased frequencies of reappearances over time, *F* (1, 6) = 25.55, *p* = *25.55*, η^2^_p_ = 0.81. Reappearances were more frequent at the 400 ms time window (Mean = 52%, SD = 12%) relative to the 300 ms time window (Mean = 43%, SD = 11%), *t*(6) = −5.05, *p* = 0.002.

**Figure 3:**
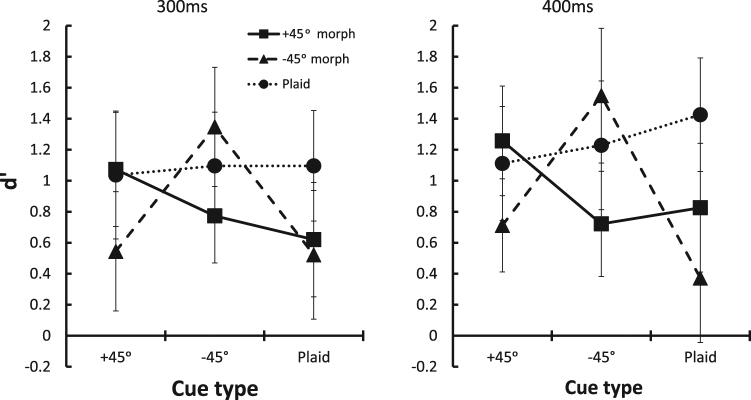
d’ as a function of morph and cue type at the 300 ms and 400 ms time windows. Data points represent means across observers (N = 7), and error bars represent the standard error of the mean.

To control for the possible effect of target-dependent spontaneous-reappearance, the results were converted into d’^12^. Cue-driven reappearances were considered as Hits and spontaneous reappearances as False-Alarms (computing d’ by subtracting the Z scores of the induced reappearances from the Z scores of spontaneous reappearances). By using this measure, we also control for the effect of the morph on the occurrences of spontaneous reappearances. Here, high d’ scores represent the high effectiveness of the cue, which does not depend on spontaneous or morph-induced reappearances. This analysis revealed a pattern of results (Fig. 3) similar to that observed with the frequencies of reappearances, despite a larger inter-observer variability in cue sensitivity (as indicated by the larger SEs in Fig. 3). An exception is the results of the 400 ms time window with the +45° morphed target, which showed only a marginally significant difference (p < 0.06) between the same-orientation cue (+45°) and the plaid cue. The d’ analysis, by capturing the difference between cue-driven reappearances and spontaneous reappearances, revealed the relatively high cue-sensitivity of the plaid target, showing no cue selectivity, in agreement with our previous results^9^.

## Discussion

In the current work we used the MIB paradigm to test the reappearance of perceptually invisible stimuli upon the presentation of a visible cue^10^. After observing similarity-dependent interactions between the aware and the unaware states^9^, we were encouraged to investigate whether the interactions are memory driven, that is, dependent on the representation of the invisible target during the time of suppression. We suggested that selective interactions between the modified target representation and the cue imply that visual representations are continuously processed and can be updated during perceptual suppression. Here a plaid target was morphed into a Gabor patch, once the observers reported its disappearance. Overall, our findings indicate real-time interactions between the perceptually visible and the perceptually invisible stimuli, demonstrating that visual representations can be modified even in the absence of conscious perception, in agreement with previous reports^8^. The cues induced the reappearance of the target, depending on its properties during perceptual suppression; a morphed Gabor target reappeared more frequently in the presence of a Gabor cue with the same orientation than with a plaid cue; the latter exhibited behavior that was not significantly different from that of spontaneous reappearances. These selective interactions, between the cue and the modified representation of the target, indicate high sensitivity to unperceived changes in visual stimulation even without conscious perception. Furthermore, the results suggest that the interactions do not depend on a past representation, as perceived before perceptual suppression, but instead indicate ongoing visual processes across awareness states.

The results showed that an invisible plaid target efficiently and uniformly reappeared with component cues and a plaid cue, but that a plaid cue produced target-specific reappearances. These results are in agreement with our previous findings in showing non-symmetric interactions between the perceived and the unperceived stimuli^9^; an invisible object target was induced to reappear by its visible component cues and the object cue; however a visible object cue was not effective with component targets. It was proposed that out of awareness, a plaid is represented at different levels of abstractions, including both features and objects. Therefore, we suggested that visual representations of objects and their components are formed in the absence of conscious experience, whereas a conscious perception is confined to object representations. Taken together, the previous and the current results indicate that the two awareness stats are well distinguished with respect to visual features defined at early stages of visual processing, but not with respect to objects. Thus, whereas in the absence of conscious experience several levels of visual abstractions exist, in its presence only one representation is effective. We suggest here that this dominance, obtained with conscious awareness, is required for further decision making and action.

The involvement of conscious perception in the formation of visual representations is the focus of research on visual awareness^13–16^. In a MIB study, Mitroff & Scholl^8^ showed that perceptual groups can be formed outside of awareness, through spatial integration. Changes made in the grouping relations of unperceived stimuli, during MIB suppression, affect their reappearance. In the current work we changed the properties of a single stimulus, reducing an object into one of its components. Moreover, here the representations of the invisible were tested implicitly, by showing interactions across awareness states between an invisible stimulus and its visible context.

In the current study we showed a real-time interaction between a perceptually suppressed stimulus and its visible context. By changing the properties of an unperceived object during perceptual suppression, we showed a similarity-dependent interaction between the visible stimulus and the modified representation of the invisible stimulus. Our findings show that there is high sensitivity to unperceived changes in representation, indicating that visual representations can be modified without conscious perception.
